# Stevens‐Johnson Syndrome due to COVID‐19 vaccination

**DOI:** 10.1002/ccr3.5099

**Published:** 2021-11-16

**Authors:** Parvin Mansouri, Reza Chalangari, Katalin Martits‐Chalangari, Nikoo Mozafari

**Affiliations:** ^1^ Skin and Stem Cell Research Center Tehran University of Medical Science Tehran Iran; ^2^ Medical Laser Research Centers Academic Center for Education, Culture and Research Tehran University of Medical Sciences Tehran Iran; ^3^ Kassir Dermatology Dallas TX USA; ^4^ Skin Research Center Shahid Beheshti University of Medical Sciences Tehran Iran; ^5^ Department of Dermatology Loghman Hakim Hospital Shahid Beheshti University of Medical Sciences Tehran Iran

**Keywords:** COVID‐19, Sinopharm, Stevens‐Johnson syndrome, vaccinations

## Abstract

As public COVID‐19 vaccination programs are being implemented, it is possible that more rare and serious adverse effects such as Stevens‐Johnson syndrome (SJS) and toxic epidermal necrosis (TEN) may occur.

## Graphical abstract

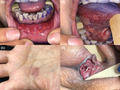

## 1 INTRODUCTION

Since the start of the COVID‐19 vaccinations, many dermatological manifestations including urticaria and morbilliform eruptions have been described. There are certain safety‐related events that, due to rarity, might be detected only during the mass vaccination programs. Here, we report a potentially serious adverse effect of COVID‐19 vaccines that have been rarely reported before.

On May 7, 2021, the World Health Organization (WHO) listed the Sinopharm COVID‐19 vaccine for emergency use.^1^ The Sinopharm vaccine is produced by the China National Biotec Group (CNBG). The Sinopharm product is an inactivated vaccine administered in two doses with a spacing of three to four weeks. The published phase 1 and 2 trial data for the inactivated Vero cell vaccines show that the most common adverse reaction was injection site pain, followed by fever, fatigue, headache, and nausea which were mild and self‐limiting; no serious adverse reactions were reported.^2^ There are certain safety‐related events that, due to rarity, might be detected only during the mass vaccination programs. Here, we report a potentially serious adverse effect of COVID‐19 vaccines that have been rarely reported before.

## 2 CASE REPORT

A 49‐year‐old woman with a background history of successfully treated breast cancer was admitted following a reaction to the second dose of COVID‐19 vaccination (.05 ml IM, COVID‐19 vaccine, Sinopharm, Beijing Bio‐Institute of Biological Products Co Ltd). On the day of vaccination, she experienced a headache, nausea, myalgia, and burning sensation in the mouth and genitalia. Over the next three days, she noted the appearance of ulcers on her lips, oral cavity, and vagina. The appearance of a single isolated rash was also noted on her left palm. The patient was complaining of odynophagia and dysuria, while she did not report any fever, vomiting, joint pain, dyspnea, or wheezing.

There was a history of similar lesions but with much less severity five days after receiving the first dose of vaccine which was resolved completely in 1 week.

Her drug history included tamoxifen, sodium valproate, and alprazolam taken with no dose changes for at least 4 years. She denied taking any new medications, supplements, or food that might have led to the reactions.

**FIGURE 1:**
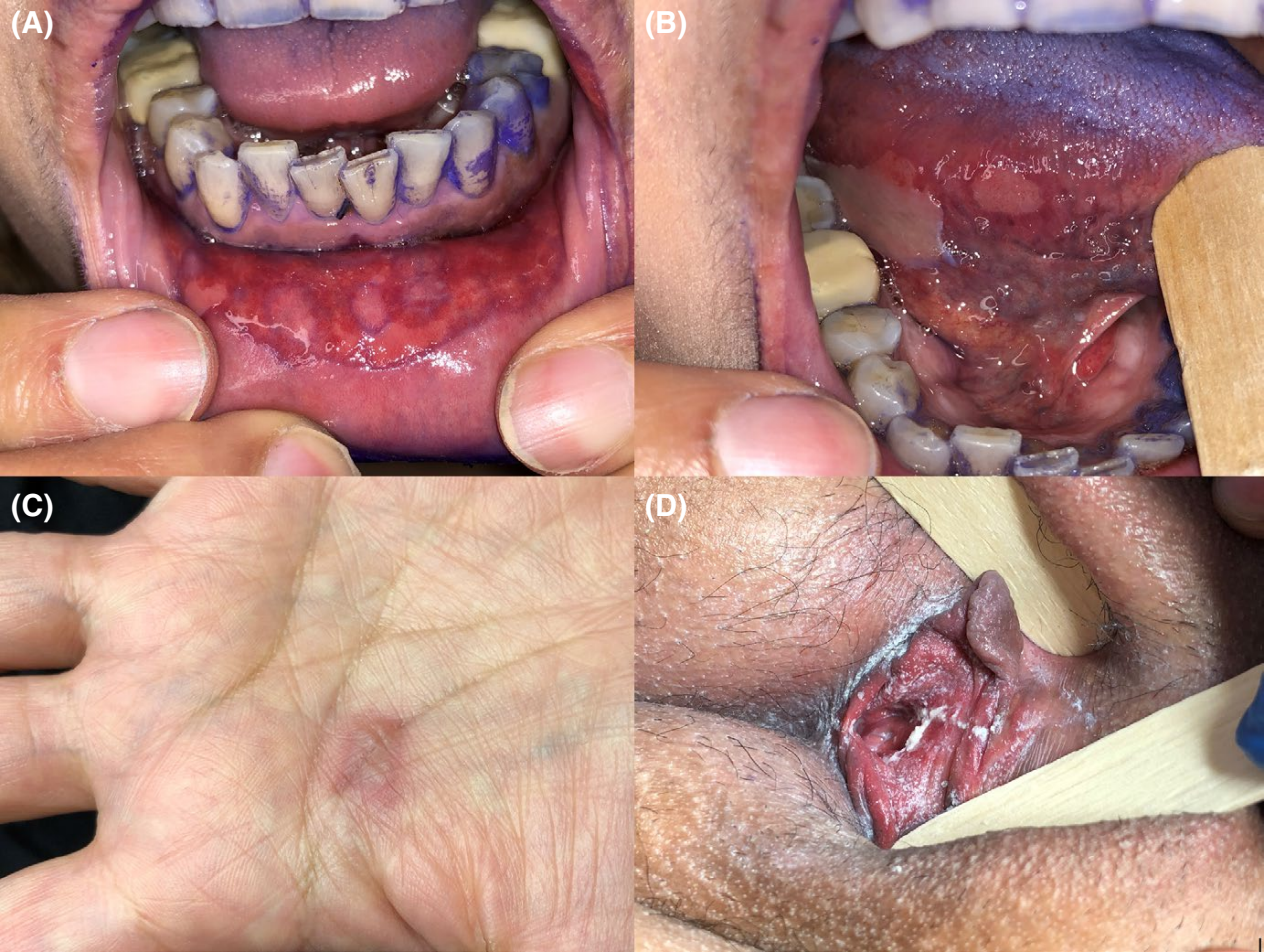
Multiple erosions on the lower lip mucosa and over the lateral surface of the tongue (A, B). An erythematous patch with a blister on the palm(C).glazed erythema of inner aspect of labia minora (D)

On examination, she looked unwell and walked slowly with difficulty. The vital signs were within normal limits and other systemic examinations were normal. There were multiple ulcerations and erosions on the bilateral buccal mucosa, lip mucosa, lower lip vermilion, and over the dorsal, lateral, and ventral surface of the tongue. On genital examination, glazed erythema and erosion of the inner aspect of labia minora around the vaginal orifice were evident. On skin examination, there was only a well‐defined circular erythematous patch with a blister on the palm (Figure 1). Cutaneous biopsy specimen showed full‐thickness epidermal necrosis, sub‐epidermal splitting, and superficial perivascular lymphocytic infiltration. The clinical and histological findings were consistent with a diagnosis of Stevens‐Johnson syndrome (SJS).

The patient was started on antihistamines (fexofenadine 180mg daily) and prednisolone (30 mg daily for a week then tapered off 10mg every week). Topical violet gentian was employed as an antiseptic for oral ulcers. Two weeks later, the mucocutaneous lesions were markedly resolved.

## 3 DISCUSSION

Since the start of the COVID‐19 pandemic, many dermatological manifestations including oral lesions have been described, Cebeci F. et al. first described the mucosal damage in COVID‐19 patients presenting with diffuse oropharyngeal erythema, petechia, and pustule formation on soft palate.^3^ Thereafter, Binois et al reported a pure mucosal case of erythema multiforme major in a COVID‐19‐positive inpatient presenting with oral and genital ulcer and conjunctivitis.^4^ It is suggested that mucosal damages in COVID‐19 patients are more likely to result from the host immune response to virus infection rather than the direct cytopathic effects of virus.^5^ Following the vaccination, the immune response against the virus is provoked, and neutralizing antibodies directed against SARS‐CoV‐2 Spike protein are produced. Then mucosal damage, similar to the COVID‐19 patient, could also be expected following vaccination. Interestingly, Azzi et al. have recently described a case of a young woman who developed diffuse, erythematous, and swollen red lesions on her buccal mucosa, tongue, gums, and palate three days after receiving the first dose of COVID‐19 vaccine (AstraZeneca ChAdOx1).^6^ In addition to a registry‐based study of cutaneous reactions after messenger RNA (mRNA)‐based COVID‐19 vaccines, observed reactions to Moderna and Pfizer vaccines, had been noted after the SARS‐CoV‐2 infection itself, including pernio/chilblains, erythromelalgia, and pityriasis‐rosea‐like exanthems.^7^

SJS is a rare immune‐mediated cutaneous reaction typically secondary to medications and less commonly to infections. Re‐exposure to a culprit agent leads to a recurrence that is usually more severe than the first episode and can be life‐threatening. It is rarely associated with vaccinations and recently three cases of SJS/TEN secondary to the COVID‐19 vaccine have been reported (Table1). Dash et al. reported SJS in a 60‐year‐old man who developed fever, oral ulceration, and skin rash 3 days after receiving the first dose of Covishield (patent product of AstraZeneca in India).^8^ Elboraey et al reported a middle‐aged female patient presenting with multiple large ulcers on buccal and labial mucosa 5 days after she had received the second dose of the COVID‐19 Pfizer vaccine.^9^ The third case was a 49‐year‐old woman who developed generalized dusky red skin eruption, extensive oral ulceration, and bilateral conjunctivitis one week after receiving the first dose of COVID‐19 Pfizer vaccine.^10^

**TABLE 1:** SJS/TEN cases reported after Covid‐19 vaccination, N/R not reported

Authors	country	Age (year)	Sex	Vaccine Type	Onset	Symptoms	Treatment	Course and Prognosis
Bakir et al.	United States	49	F	COVID‐19 Pfizer (BNT162b)	One week after the first dose	Extensive mucosal (oral, genitalia, and conjunctiva) involvement, vesiculobullous lesions and epidermal detachment over more than 30% of body surface area	Two doses of tumor necrosis factor‐alpha inhibitor (Etanercept)on the first and second days of admission	Complete healing after 22 days
Dash et al.	India	60	M	Patent product of AstraZeneca in India	3 days after the first dose	Mucosal involvement (oral and genital erosions and conjunctivitis), generalized skin rash	N/R	N/R
Elboraey et al.	Saudi Arabia	Middle‐aged	F	COVID‐19 Pfizer	5 days after the second dose	Multiple large ulcers on the oral mucosa,no skin involvement	Oral prednisolone (30 mg/d)	N/R
Mansouri et al. (Present report)	Iran	49	F	Sinopharm	3 days after the second dose	Oral and genital erosions and *a blister* on the palm	Oral prednisolone (30 mg/d), fexofenadine (180mg/d)	Markedly resolution in two weeks

In this case report, our patient experienced mild mucositis after the first dose of vaccination which was not considered a concerning sign, then when she received the second dose a more severe and faster reaction was occurred.

COVID‐19 vaccines are newly developed vaccines, and these vaccines have been developed and tested within a short period for which rare vaccines' adverse effects are relatively unrecognized. As public vaccination programs are being implemented, it is possible that more serious adverse effects such as SJS and toxic epidermal necrosis (TEN) may occur. To deal effectively with the concerns associated with risks of immunization, healthcare providers should be educated about these potential vaccine reactions and advise patients accordingly.

## CONFLICT OF INTEREST

None.

## AUTHOR CONTRIBUTIONS

**Parvin Mansouri contributed to** clinical evaluation and management of the patient and supervision of the project, editing of the final draft of the manuscript. **Reza Chalangari** and **Katalin Martits‐Chalangari** contributed to writing and editing the final draft of the manuscript. **Nikoo Mozafari** contributed to data gathering and writing the manuscript. We declare that none of the authors listed on the manuscript are employed by a government agency that has a primary function other than research and/or education. And none of the authors are submitting this manuscript as an official representative or on behalf of the government.

## CONSENT

The patient in this manuscript gave written informed consent for the publication of her case details.

## Data Availability

The data presented in this study are available on request from the corresponding author.
